# Associations of self‐reported obstructive sleep apnea with cognition and dementia risk in cognitively unimpaired middle‐aged adults

**DOI:** 10.1002/alz.71553

**Published:** 2026-06-29

**Authors:** Gabriel T. Abdelmessih, Lisa Bransby, Hannah Cummins, Melinda L. Jackson, Yen Ying Lim

**Affiliations:** ^1^ Turner Institute for Brain and Mental Health School of Psychological Sciences Monash University Clayton Victoria Australia

**Keywords:** Alzheimer's, APOE, cognition, dementia, memory, midlife, neuropsychology, OSA, sleep, vascular risk

## Abstract

**INTRODUCTION:**

Obstructive sleep apnea (OSA) is a potential risk factor for cognitive impairment and dementia; however, its contribution in midlife and interactions with *APOE* ε4 remain unclear.

**METHODS:**

Participants were 2795 cognitively unimpaired, middle‐aged adults enrolled in the Healthy Brain Project. OSA status was determined by self‐report. Cognition was assessed using the Cogstate Brief Battery, and dementia risk using the Cardiovascular Risk Factors, Ageing, and Incidence of Dementia (CAIDE) score.

**RESULTS:**

Participants with OSA demonstrated poorer memory than those without OSA, although this association was attenuated after adjusting for vascular risk. Individuals with OSA (with or without *APOE* ε4) had significantly higher CAIDE scores than those with neither risk factor. *APOE* ε4 did not moderate OSA‐cognition associations.

**DISCUSSION:**

OSA may be associated with poorer memory and greater dementia risk, irrespective of *APOE* ε4 carriage. These findings highlight the need for early OSA screening to identify individuals at elevated dementia risk.

## Highlights

- OSA was associated with poorer memory in at‐risk middle‐aged adults, though this association was attenuated after adjustment for vascular risk factors.
- OSA was also associated with elevated dementia risk, including risk driven by modifiable vascular factors such as obesity, hypertension, hypercholesterolemia, and physical inactivity.
- *APOE* ε4 did not moderate the associations of OSA with cognition or dementia risk.
- This work highlights that routine screening for OSA in midlife may help identify individuals at elevated dementia risk.

## 1 BACKGROUND

Obstructive sleep apnea (OSA) is a common sleep disorder characterized by recurrent collapse of the pharyngeal airway during sleep, causing intermittent hypoxemia and sleep fragmentation.^1^ OSA has emerged as a potential risk factor for late‐life cognitive impairment and dementia, including Alzheimer's disease (AD) dementia.^2^, ^3^, ^4^, ^5^ Pathophysiological models propose that intermittent hypoxemia and sleep fragmentation directly contribute to oxidative stress, cerebrovascular injury, and impaired glymphatic clearance of amyloid‐*β*, ultimately resulting in neuronal dysfunction and death.^6^ OSA may also indirectly result in cognitive impairment and dementia risk through its association with other established dementia risk factors, including hypertension,^7^ hypercholesterolemia,^8^ obesity,^9^ and physical inactivity.^10^

Importantly however, the relationship between OSA and the risk it poses on cognitive impairment and dementia remains equivocal, likely resulting from inconsistent findings to date. While studies in clinical populations more consistently support a relationship between OSA, cognitive impairment, and dementia risk,^11^, ^12^, ^13^ findings from community‐based samples have been mixed. Some studies have found OSA to be associated with deficits in multiple cognitive domains and increased dementia risk,^3^, ^14^, ^15^, ^16^, ^17^, ^18^, ^19^ whereas others have reported no such relationships.^20^, ^21^, ^22^, ^23^ These discrepancies may reflect methodological differences, including sample characteristics (e.g., clinic‐based samples including individuals with more severe OSA), inadequate control for potential confounders (e.g., vascular risk factors), and the use of neuropsychological tests that lack sensitivity to early insults to the central nervous system. Thus, further research is warranted to clarify whether OSA is associated with cognition and dementia risk, particularly within community‐based samples.

An additional consideration in the relationship between OSA and cognitive impairment is the presence of the apolipoprotein E (*APOE*) ε4 allele, the strongest known genetic risk factor for sporadic AD.^24^ In older adults, carriage of even one copy of the *APOE* ε4 allele is associated with greater susceptibility to cerebrovascular disease and increased amyloid‐*β* deposition.^25^, ^26^, ^27^, ^28^, ^29^, ^30^ Emerging research suggests that in middle‐aged and older ε4 carriers, OSA is more strongly related to poorer cognitive function than in non‐carriers.^14^, ^31^, ^32^, ^33^, ^34^, ^35^ However, the additive or synergistic influence of OSA and *APOE* ε4 on cognitive function and dementia risk remain unclear, particularly in middle‐aged adults (e.g., 40–70 years). Investigating these relationships in midlife, before the onset of frank cognitive impairment or significant neuropathological changes, is crucial, as this is when dementia prevention strategies may be most efficacious.^36^

The first aim of this study was to investigate the associations of OSA with cognition and dementia risk, operationalized using the Cardiovascular Risk Factors, Ageing and Incidence of Dementia (CAIDE) risk score, in a large, community‐based sample of cognitively unimpaired, middle‐aged adults enriched for a family history of dementia. It was hypothesized that individuals with OSA would exhibit poorer cognition and greater dementia risk compared to those without OSA. The second aim was to explore the nature and extent to which *APOE* ε4 carriage modified these associations.

## 2 METHODS

### 2.1 Participants

**FIGURE 1 alz71553-fig-0001:**
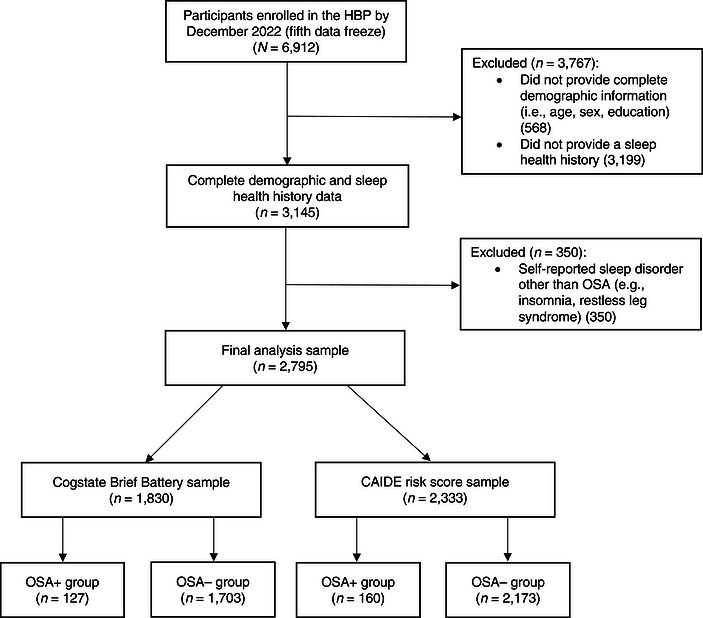
Participant selection process.

This cross‐sectional study included baseline data for 2795 participants enrolled in the Healthy Brain Project (HBP; healthybrainproject.org.au; Figure 1). The HBP is a prospective, community‐based online cohort of cognitively unimpaired adults, enriched with a family history of dementia.^37^ The recruitment and enrolment procedures for the HBP have been comprehensively detailed previously.^37^ Briefly, participants were required to be aged 40–70 years, reside in Australia, and be fluent in English to complete the online assessments. Exclusion criteria included self‐reported cognitive impairment, neurological conditions (e.g., acquired brain injury, multiple sclerosis), neurodegenerative diseases (e.g., dementia, Parkinson's disease), or any use of Therapeutic Goods Administration‐approved medications for AD (e.g., donepezil, memantine).

The HBP was approved by the Monash University Human Research Ethics Committee, and all participants provided informed consent via the online platform before study commencement. Data were collected between November 2016 and December 2024, with the current study including data collected up until the fifth data freeze (December 2022). This report adheres to the Strengthening the Reporting of Observational Studies in Epidemiology (STROBE) guidelines.^38^

**RESEARCH IN CONTEXT**

1. **Systematic review**: The authors reviewed the literature using traditional sources (e.g., PubMed). While obstructive sleep apnea (OSA) OSA has been associated with cognitive impairment and dementia, findings in community‐based samples have been inconsistent. Evidence also suggests that OSA interacts with *APOE*ε4 on cognitive function, although results are mixed.
2. **Interpretation**: In a large, community‐based sample of cognitively unimpaired, middle‐aged adults, OSA was associated with poorer memory and greater dementia risk. These associations were partly explained by vascular risk factors and were not moderated by *APOE* ε4 status. These findings suggest that the contribution of OSA to cognitive function and dementia risk in midlife may be independent of *APOE* ε4 status.
3. **Future directions**: Prospective studies are needed to determine whether OSA accelerates cognitive decline over time and whether *APOE* ε4 modifies these trajectories as individuals age. Randomized controlled trials are also needed to determine whether treating OSA in midlife can reduce vascular burden and ultimately mitigate dementia risk.

### 2.2 Demographic characteristics

Participants self‐reported their date of birth, sex, height, and weight (used to calculate body mass index [BMI]), years of education, ethnicity, and medical history (including history of hypertension, hypercholesterolemia, and sleep disorders). Participants also completed the Hospital Anxiety and Depression Scale (HADS),^39^ to determine depressive and anxiety symptoms; the Epworth Sleepiness Scale (ESS),^40^ to determine levels of daytime sleepiness; the Insomnia Severity Index (ISI),^41^ to assess insomnia symptoms and severity; the Pittsburgh Sleep Quality Index (PSQI),^42^ to assess sleep quality;^42^ and the International Physical Activity Questionnaire (IPAQ),^43^ to determine physical activity levels.[Fig alz71553-fig-0001]

### 2.3 OSA status

Participants who self‐reported a clinical diagnosis of OSA were assigned to the OSA+ group, while those who did not comprised the OSA− group. Participants in the OSA+ group also indicated whether they were currently receiving treatment for their OSA (treated/untreated).

### 2.4 Cognition

Participants completed the Cogstate Brief Battery, which has been validated for unsupervised, online administration.^37^, ^44^, ^45^ As described previously,^46^, ^47^ the battery comprises four subtests: Detection, Identification, One‐Back, and One‐Card Learning. Briefly, Detection measured psychomotor processing speed through a simple reaction time task; Identification evaluated visual attention via a choice reaction time paradigm; One‐Back assessed working memory using a one‐back task; and One‐Card Learning evaluated visual learning through a continuous recognition paradigm within a pattern separation model. Reaction time (milliseconds) was the primary outcome for Detection and Identification, which was log_10_‐transformed and reversed so that higher scores reflected better performance. Accuracy was the primary outcome for One‐Back and One‐Card Learning, which was arcsine square root transformed, with higher scores reflecting better accuracy. All raw scores were standardized using the sample mean and standard deviation. Composites for attention and memory were computed, respectively, by averaging standardized Detection and Identification scores and standardized One‐Back and One‐Card Learning scores. These composites have demonstrated sensitivity to mild cognitive impairment (MCI) and AD, with studies suggesting greater sensitivity compared to individual subtests.^46^, ^47^, ^48^ Distribution of *z*‐scores for each cognitive composite are shown in Figure S1.

### 2.5 Dementia risk

The CAIDE risk score has been previously validated as an effective tool for predicting late‐life cognitive impairment and dementia in midlife.^49^, ^50^ The score (without *APOE* ε4) incorporates seven risk factors: age, years of education, sex, BMI (kg/m^2^), physical activity, history of hypercholesterolemia, and history of hypertension (Table S1). Total scores ranged from 0 to 15, with higher scores indicating greater dementia risk. To isolate dementia risk attributable to vascular risk factors both associated with OSA and readily modifiable, a modified version of the CAIDE risk score was also computed, including only measures of BMI, physical activity, history of hypercholesterolemia, and history of hypertension.^51^, ^52^, ^53^, ^54^ Modified CAIDE risk scores ranged from 0 to 7, with higher scores reflecting greater vascular risk burden.

### 2.6 *APOE* genotyping

Genotek Oragene (OG‐500) 2 mL saliva kits were mailed to participants who completed at least 80% of assessments on the larger HBP online platform.^37^ Returned saliva samples were forwarded to GenoFIND Services Laboratory (Salt Lake City, USA), where they were processed to extract targeted SNPs, including *APOE* (rs429358, rs7412), using the TaqMan GTXpress Master Mix (Life Technologies) methodology according to manufacturer instructions.

### 2.7 Statistical analyses

Analyses were performed in R (version 4.4.0). Demographic and health characteristics were compared between the OSA+ and OSA− groups using *t*‐tests for continuous variables and *χ* ^2^ tests for categorical variables.

To examine between‐group differences in cognition, dementia risk, and modifiable vascular dementia risk, a series of analyses of covariance (ANCOVAs) were performed, with OSA group (OSA−, OSA+) as a fixed factor. Covariates included age (years), sex (male/female), education (years), and OSA treatment status (yes/no). For analyses involving the original CAIDE risk score, age, sex, and education (years), were not included as covariates, as these variables are components of the score itself.

To determine whether these associations were modified by *APOE* ε4 carriage, OSA group × *APOE* ε4 status interaction terms were added to each model. Planned comparisons were conducted to quantify: (1) the individual contribution of OSA in the absence of ε4 (OSA+/ε4− versus OSA−/ε4−), (2) the individual contribution of *APOE* ε4 in the absence of OSA (OSA−/ε4+ versus OSA−/ε4−), and (3) whether the combined risk of OSA and *APOE* ε4 (OSA+/ε4+) was associated with poorer cognition and greater dementia risk compared to each individual risk group (OSA+/ε4−, OSA−/ε4+, OSA−/ε4−).

Estimated marginal means were calculated for each group for all comparisons, with the magnitude of difference between groups quantified using Cohen's *d* effect sizes with 95% confidence intervals (CIs). False discovery rate (FDR) correction for multiple comparisons was applied using the Benjamini−Hochberg method.^55^ Statistical significance was set to *p* < 0.05 for all analyses, except for interaction effects, which were considered significant at *p* < 0.10 due to their typically lower statistical power relative to main effects.

#### 2.7.1 Sensitivity analyses

In a first sensitivity analysis, to examine whether associations differed by OSA treatment status, the primary ANCOVA models were repeated with OSA group coded according to treatment status (OSA−, untreated OSA+, and treated OSA+). OSA treatment status was excluded as a covariate in these models. In a second sensitivity analysis, we examined whether cumulative vascular risk, indexed by the modified CAIDE risk score, accounted for the associations between OSA and cognition, given vascular risk factors commonly co‐occur with OSA, and are associated with cognitive function and increased dementia risk.^8^, ^56^, ^57^, ^58^ First, the modified CAIDE risk score was included as an additional covariate in the primary ANCOVA models. A formal mediation analysis was also conducted to determine whether the modified CAIDE risk score mediated any significant between‐group differences in cognition. Indirect effects were tested using a 95% bias‐corrected CI derived from 1000 bootstrap samples.

## 3 RESULTS

### 3.1 Demographic characteristics

Of the 2795 participants (mean [SD] age = 56.44 [7.09]; 76% female), 195 (7%) reported a diagnosis of OSA (OSA+) and 2600 (93%) did not (OSA−). The demographic and health characteristics of the sample stratified by OSA group are summarized in Table 1. *APOE* ε4 carriers comprised 33.5% of the sample, and 55.4% had a first‐ or second‐degree family history of dementia. On average, compared to the OSA− group, the OSA+ group was older, more likely to be men, had a higher BMI, fewer years of education, greater depression and anxiety symptoms, and a higher proportion of vascular risk factors. The OSA+ group also reported greater daytime sleepiness, poorer sleep quality, more severe insomnia symptoms, and more frequently endorsed both stopping breathing and snoring or coughing loudly during sleep (28% vs. 6%). Of the OSA+ group, 58.9% reported current treatment for their OSA.

**TABLE 1:** Demographic characteristics.

	Mean (SD) or No. (%)			
Characteristics	Total sample (*N* = 2795)	OSA– group(*n* = 2600)	OSA+ (*n* = 195)	*p*‐Value
Age, years	56.44 (7.09)	56.28 (7.12)	58.49 (6.46)	<0.001
Female	2123 (76.0%)	2014 (77.5%)	109 (55.9%)	<0.001
Education, years	16.11 (3.43)	16.16 (3.41)	15.46 (3.68)	0.01
Caucasian	2296 (82.2%)	2141 (82.4%)	155 (79.5%)	0.34
*APOE* ε4 carrier^*^	706 (33.5%)	650 (33.2%)	56 (37.8%)	0.50
First‐ or second‐degree family history of dementia	1495 (55.4%)	1390 (55.3%)	105 (57.1%)	0.64
BMI, kg/m^2^	26.62 (5.52)	26.34 (5.43)	30.31 (5.45)	<0.001
Hypertension	636 (22.8%)	545 (21.0%)	91 (46.7%)	<0.001
Hypercholesterolemia	827 (29.6%)	739 (28.4%)	88 (45.1%)	<0.001
HADS^†^				
Anxiety, total score	4.13 (3.36)	4.08 (3.34)	4.73 (3.64)	0.01
Depression, total score	2.54 (3.11)	2.45 (3.03)	3.80 (3.85)	<0.001
Physical activity				0.08
Low	263 (10.9%)	238 (10.6%)	25 (15.2%)	
Moderate	1028 (42.6%)	954 (42.4%)	74 (45.1%)	
High	1122 (46.5%)	1057 (47.0%)	65 (39.6%)	
PSQI, total score^‡^	5.38 (3.36)	5.45 (3.40)	6.45 (3.82)	<0.001
ESS, total score	5.22 (3.69)	5.10 (3.63)	6.87 (4.15)	<0.001
ISI, total score^¶^	6.26 (5.02)	6.15 (4.95)	7.67 (5.72)	<0.001

Abbreviations: APOE, apolipoprotein E; BMI, body mass index; ESS, Epworth Sleepiness Scale; HADS, Hospital Anxiety and Depression Scale; ISI, Insomnia Severity Index; OSA, obstructive sleep apnea; PSQI, Pittsburgh Sleep Quality Index.

* Sample size of participants with available APOE genotyping data = 2106.

^†^ Score range, 0 to 21 points for each subscale (anxiety, depression), with scores > 8 indicating clinically significant symptoms.

^‡^ Score range, 0 to 21, with scores > 5 indicating poor sleep quality.

^§^ Score range, 0 to 24 points, with scores > 10 indicating excessive daytime sleepiness.

^¶^ Score range, 0 to28 points, with scores > 10 clinically significant insomnia symptoms.

### 3.2 OSA and cognition

After adjustments for age, sex, education, and OSA treatment, participants in the OSA+ group demonstrated poorer performance on the memory composite compared to those in the OSA− group (Table 2). The magnitude of this difference was moderate by convention. No between‐group differences were observed in performance on the attention composite.

**TABLE 2:** Associations of OSA with cognition and dementia risk.

	ANCOVA		EMM (SE)		OSA+ versus OSA–	
Parameter	*β* (SE)	*p*‐Value	OSA–	OSA+	Cohen's *d* (95% CI)	*p‐*Value
Attention composite	−0.13 (0.11)	0.52	0.02 (0.02)	−0.04 (0.08)	−0.06 (−0.25, 0.12)	0.49
Memory composite	−0.27 (0.10)	0.01	−0.00 (0.02)	−0.17 (0.07)	−0.22 (−0.04, −0.40)	0.03
CAIDE risk score	1.62 (0.29)	<0.001	5.08 (0.19)	7.04 (0.05)	0.84 (0.68, 1.00)	<0.001
Modified CAIDE risk score	0.87 (0.21)	<0.001	1.50 (0.04)	2.72 (0.14)	0.72 (0.56, 0.89)	<0.001

Abbreviations: ANCOVA, analysis of covariance; CAIDE, Cardiovascular Risk Factors, Ageing and Incidence of Dementia; CI, confidence interval; EMM, estimated marginal mean; OSA, obstructive sleep apnea; SE, standard error.

*Note*: Models adjusted for age, sex, education, and OSA treatment where appropriate. *p*‐Values are corrected for multiple comparisons. Sample sizes: Attention composite, *N* = 1830 (OSA− = 1703, OSA+ = 127); Memory composite, *N* = 1791 (OSA− = 1666, OSA+ = 125); CAIDE risk scores, *N* = 2333 (OSA− = 2173, OSA+ = 160).

### 3.3 OSA and dementia risk

Participants in the OSA+ group had a higher original CAIDE risk score compared to those in the OSA− group after adjustment for OSA treatment (Table 2). The magnitude of this difference was large by convention. These results were comparable when using the modified CAIDE risk score, although the magnitude of this difference was moderate by convention.

### 3.4 Modification by *APOE* ε4 carriage

**FIGURE 2:**
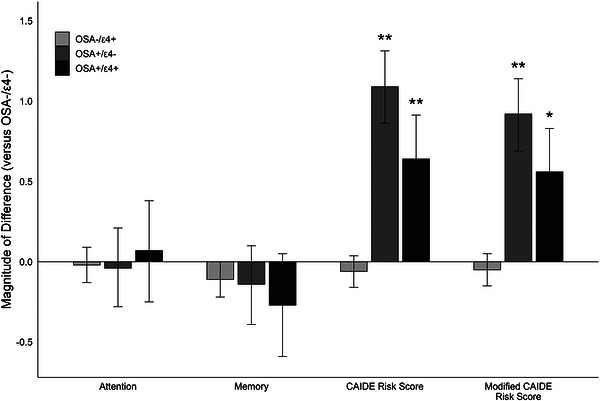
The magnitude of difference in attention, memory, dementia risk, and modifiable vascular dementia risk between *APOE* ε4 non‐carriers without OSA and those with *APOE* ε4 only, OSA only and OSA and *APOE* ε4. Error bars represent 95% confidence intervals. ^*^ Indicates *p *< 0.01. ^**^ Indicates *p* < 0.001. APOE, apolipoprotein E; OSA, obstructive sleep apnea.

**FIGURE 3:**
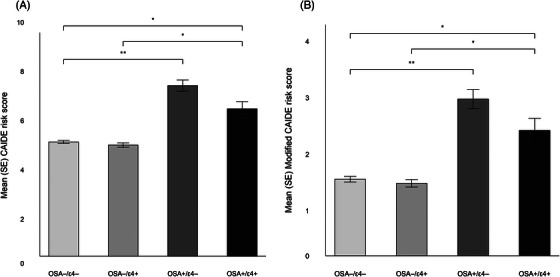
Bar plots indicate mean (A) CAIDE risk scores and (B) modified CAIDE risk scores across OSA/*APOE* ε4 groups. Error bars represent standard error. ^*^ Indicates *p *< 0.01. ^**^ Indicates *p* < 0.001. APOE, apolipoprotein E; CAIDE, Cardiovascular Risk Factors, Ageing and Incidence of Dementia; OSA, obstructive sleep apnea.

Significant interactions between OSA and *APOE* ε4 were observed for the original CAIDE risk score (*F*_2‐2326 _= 5.99; *p* = 0.01) and modified CAIDE risk score (*F*_2‐2323 _= 3.50; *p* = 0.06). Pairwise comparisons indicated that, compared to participants with neither risk factor (OSA−/ε4−), those with OSA only (OSA+/ε4−) and those with both OSA and *APOE* ε4 (OSA+/ε4+) had significantly higher original and modified CAIDE risk scores, while those with *APOE* ε4 only (OSA−/ε4+) did not (Table 3 and Figure 2). Participants with both OSA and *APOE* ε4 had higher original and modified CAIDE scores than those with *APOE* ε4 only (OSA−/ε4+), but not compared to those with OSA only (OSA+/ε4−) (Figure 3).

**TABLE 3:** Differences in cognition and dementia risk by OSA and *APOE* ε4 status.

Parameter	OSA–/ε4– versus OSA–/ε4+			OSA–/ε4– versus OSA+/ε4–			OSA–/ε4– versus OSA+/ε4+			OSA+/ε4+ versus OSA–/ε4+			OSA+/ε4+ versus OSA+/ε4–		
	*β* (SE)	Cohen's *d* (95% CI)	***p*‐Value**	*β* (SE)	Cohen's *d* (95% CI)	***p‐*Value**	*β* (SE)	Cohen's *d* (95% CI)	***p‐*Value**	*β* (SE)	Cohen's *d* (95% CI)	***p‐*Value**	*β* (SE)	Cohen's *d* (95% CI)	***p‐*Value**
Attention composite	−0.016 (0.05)	−0.02 (‐0.13, 0.10)	0.99	0.03 (0.11)	−0.04 (−0.28, 0.21)	0.99	0.06 (0.14)	0.07 (−0.26, 0.38)	0.99	−0.07 (0.14)	0.08 (−0.24, 0.41)	0.99	0.09 (0.17)	0.10 (−0.29, 0.50)	0.99
Memory composite	−0.08 (0.04)	−0.11 (−0.22, 0.00)	0.99	0.11 (0.1)	−0.14 (−0.39, 0.10)	0.99	−0.21 (0.13)	−0.27 (−0.59, 0.05)	0.99	0.13 (0.13)	−0.16 (−0.49, 0.16)	0.99	−0.10 (0.16)	−0.13 (−0.52, 0.27)	0.99
CAIDE risk score	−0.14 (0.12)	−0.06 (−0.16, 0.04)	0.99	−2.52 (0.26)	1.09 (0.86, 1.31)	<0.001	1.50 (0.33)	0.64 (0.36, 0.91)	<0.001	−1.61 (0.33)	0.70 (0.41, 0.98)	<0.001	−1.05 (0.41)	−0.45 (−0.80, −0.10)	0.29
Modified CAIDE risk score	−0.08 (0.09)	−0.05 (−0.15, 0.05)	0.99	−1.55 (0.19)	0.92 (0.69, 1.14)	<0.001	0.94 (0.24)	0.56 (0.28, 0.83)	<0.01	−1.02 (0.25)	0.60 (0.32, 0.89)	<0.01	−0.61 (0.30)	−0.36 (−0.71, −0.01)	0.99

Abbreviations: APOE, apolipoprotein E; CAIDE, Cardiovascular Risk Factors, Ageing and Incidence of Dementia; CI, confidence interval; OSA, obstructive sleep apnea; SE, standard error.

*Note*: Models adjusted for age, sex, education, and OSA treatment where appropriate. *p‐*Values are corrected for multiple comparisons.

No significant OSA × *APOE* ε4 interactions were observed for the attention composite (*F*_2‐1820_ = 1.73; *p* = 0.24) and memory composite (*F*_2‐1781_ = 1.30; *p* = 0.27), and planned comparisons revealed no significant group differences in attention or memory performance across OSA/*APOE* ε4 subgroups (Table 3 and Figure 2).

### 3.5 Sensitivity analyses

When OSA status was further stratified by treatment, poorer memory performance was observed among individuals with untreated OSA compared with those without OSA, while those with treated OSA did not differ significantly from the OSA− group. No significant differences in attention performance were observed for either untreated OSA or treated OSA relative to the OSA− group. Both untreated and treated OSA were associated with higher original and modified CAIDE risk scores compared with those without OSA (Table S2).

After additional adjustment for the modified CAIDE risk score, the association between OSA and memory performance was no longer significant (*β* [SE] = −0.18 [0.11]; *p *= 0.16), and the effect size was reduced to small (*d* = 0.14; 95% confidence interval [CI], −0.06 to 0.34). The association between OSA and attention performance remained nonsignificant (*β* [SE] = −0.07 [0.12]; *p *= 0.91; *d* = 0.01; 95% CI, −0.18–0.21). In the mediation model for memory, participants with OSA had significantly higher modified CAIDE risk scores compared to those without OSA (path *a*: *β*  =  0.83; 95% CI, 0.36 to 1.30; *p*  < 0.001), but modified CAIDE risk scores were not significantly associated with memory performance (path *b*: *β*  =  −0.02; 95% CI, −0.05 to 0.01; *p*  =  0.17). The indirect effect showed modified CAIDE risk scores did not mediate the association between OSA and memory performance (*β*  =  −0.01; 95% CI, −0.04 to 0.01; *p*  =  0.21).

## 4 DISCUSSION

The hypothesis that participants with OSA would have poorer cognition was partially supported. Participants with OSA demonstrated poorer memory, with the magnitude of this difference moderate by convention. No between‐group differences in attention were observed. Previous studies have reported poorer memory performance in individuals with OSA compared to those without OSA^59^, ^60^, ^61^; however, most studies to date have not accounted for the role of vascular risk in these associations.^22^, ^62^ Given that vascular risk factors such as obesity, hypertension, and hypercholesterolemia frequently co‐occur with OSA,^56^ and are themselves associated with memory impairment,^58^ it was important to determine whether OSA was independently associated with cognition beyond its vascular burden. In the current study, while the association between OSA and memory was attenuated after adjusting for vascular risk, a formal mediation analysis did not support a direct or indirect effect of vascular risk factors on memory. These results suggest that, although vascular risk might explain some of the association between OSA and poor memory in midlife, the association may not be fully mediated by vascular burden and may instead reflect underlying vulnerability that modifies susceptibility to OSA‐related cognitive effects. Notably, sensitivity analyses further indicated that poorer memory performance was evident primarily among individuals with untreated OSA, whereas individuals receiving treatment did not differ significantly from those without OSA. Although these findings should be interpreted cautiously given the cross‐sectional design and reliance on self‐reported binary treatment status, they are consistent with the possibility that effective OSA management may attenuate the impact of OSA on memory, even in the presence of elevated vascular risk. Thus, it is likely that nonvascular mechanisms, such as intermittent hypoxemia‐related medial temporal lobe atrophy,^63^ also partly underlie the association between OSA and poor memory.

The hypothesis that OSA would be associated with greater dementia risk was also supported. Compared to those without OSA, individuals with OSA showed significantly higher dementia risk, as measured by the CAIDE risk score, with the magnitude of this difference large by convention. This finding is consistent with prospective studies that show individuals with OSA are more likely to develop cognitive impairment,^2^, ^3^ and extend prior work by demonstrating that this association is evident even in a relatively young, cognitively unimpaired sample. Notably, the association remained significant when the CAIDE risk score was restricted to modifiable vascular factors (i.e., hypertension, hypercholesterolemia, physical inactivity, obesity). This observation is consistent with prior research by our group^64^, ^65^ and others^66^ that report a link between OSA and the cumulative burden of vascular risk factors, and supports broader literature implicating OSA in the development of cardiovascular and cerebrovascular risk.^7^, ^56^, ^67^, ^68^, ^69^ The mechanisms underlying this relationship are well‐documented; intermittent hypoxia and sleep fragmentation may activate the sympathetic activity and renin‐angiotensin‐aldosterone system, leading to endothelial dysfunction, increased arterial stiffness, and chronic inflammation, all of which likely promote the development of vascular risk factors, such as hypertension.^69^ Importantly, elevated vascular dementia risk was observed in both treated and untreated OSA groups compared with those without OSA, suggesting that an OSA diagnosis in midlife may reflect an accumulated burden of vascular risk factors that may not be fully attenuated cross‐sectionally by treatment. Taken together, these findings add further support to the hypothesis that vascular mechanisms contribute to the association between OSA and late‐life cognitive impairment and dementia.

*APOE* ε4 carriage did not moderate the relationship between OSA and cognition in the current sample. Specifically, individuals with both OSA and *APOE* ε4 did not have poorer memory than the other subgroups, and the magnitude of group differences was small to moderate by convention (*d*'s = 0.11–0.27). These findings contrast with previous cross‐sectional studies that have reported an interaction between OSA and *APOE* ε4 on cognition in middle‐aged adults across both community‐based and clinical samples.^14^, ^31^, ^33^ There are several potential reasons for this discrepancy. First, *APOE* ε4‐related cognitive deficits are more consistently observed later in life.^70^ In middle‐aged adults, ε4‐related cognitive deficits have primarily been observed in *APOE* ε4 homozygotes rather than heterozygotes.^71^ Given the relatively young age of the current sample and the small number of *APOE* ε4 homozygotes (i.e., *n *= 74), it is likely that the current study was underpowered to adequately test OSA × *APOE* ε4 interactions on cognition at cross‐section. Second, the interactive effect of OSA and *APOE* ε4 on cognition has been primarily observed in cases of severe OSA.^31^, ^32^, ^33^, ^34^, ^35^ As HBP participants were required to self‐report their OSA status, information on OSA severity (e.g., Apnea‐Hypopnea Index, which can only be determined through polysomnography) was unavailable. The current sample also included participants who were already undergoing OSA treatment (58.9%), likely resulting in a sample with milder disease severity overall compared to previous studies.

While individuals with both OSA and *APOE* ε4 exhibited elevated dementia risk, those with OSA alone showed the greatest dementia risk. Conversely, *APOE* ε4 carriers without OSA did not differ from those with neither risk factor. This pattern is consistent with the notion that, in midlife, the vascular and cerebrovascular effects of *APOE* ε4 have not yet manifested fully, and instead accumulate gradually and become more apparent in later life.^29^, ^72^, ^73^ In cognitively unimpaired older adults, *APOE* ε4 and vascular risk have both been independently associated with greater hippocampal atrophy and episodic memory decline,^54^ while other work has shown *APOE* ε4 moderates the association between vascular risk factors and tau pathology.^74^ When considered together, vascular and genetic risk may operate independently earlier in the disease course but increasingly converge as age and pathology accumulate. Thus, while the present cross‐sectional findings suggest an early and independent contribution of OSA to dementia risk and vascular burden, it remains possible that the additive or synergistic effects of OSA and *APOE* ε4 emerge over time. Future studies will need to determine whether interactions between OSA and *APOE* ε4 on cognition and dementia risk are more evident in individuals who are older, have greater pathological burden (e.g., amyloid‐*β*, tau, cerebrovascular disease), or have more severe OSA. It will also be important to clarify whether elevated dementia risk in midlife OSA is associated with subsequent cognitive decline, and whether *APOE* ε4 moderates these trajectories.

### 4.1 Strengths and limitations

An important strength of this study was the large, well‐characterized, community‐based sample, over half of whom reported a family history of dementia. Prior studies have largely relied on sleep clinic samples,^75^ which may be biased toward individuals with more severe symptomatology and greater comorbidity burden and overall dementia risk. Therefore, our community‐based cohort may offer better insight into a broader spectrum of OSA severity and its associated risk profile in community‐dwelling adults. This enhances the generalizability of the current findings to the broader population, which is particularly relevant for population‐level dementia prevention strategies and secondary prevention trials (e.g., BetterBrains,^76^ Maintain Your Brain^77^). Further, as late‐life OSA may also represent symptoms of incipient dementia,^78^ the examination of these associations in midlife reduces the likelihood that the results of the current study reflect reverse causation.

There are also several limitations that should be considered. First, OSA status was determined by self‐reported diagnosis, which may have resulted in misclassification, especially given OSA is frequently underdiagnosed.^79^ Although the proportion of OSA cases in this sample (i.e., 7%) is comparable to previous population estimates for middle‐aged adults (e.g., 9%),^80^ some individuals with undiagnosed OSA were likely included in the OSA− group, which would be expected to bias associations toward the null. The reliance on self‐report also precluded examination of objective markers of OSA severity (e.g., Apnea‐Hypopnoea Index, hypoxemic burden). Nonetheless, this approach may have preferentially captured individuals with more symptomatic OSA sufficient to prompt clinical diagnoses.^81^ Second, detailed information regarding OSA treatment type, duration, and adherence was unavailable, limiting further inferences regarding the potential modifying effects of treatment. Third, while the Cogstate Brief Battery is validated and sensitive to early AD‐related cognitive changes,^46^, ^47^, ^48^ it is not as comprehensive as a full neuropsychological assessment. This may have also limited the ability to detect subtle OSA− or *APOE* ε4‐related impairments in other cognitive domains (e.g., verbal memory, executive function). Finally, owing to the cross‐sectional design, conclusions regarding the temporal relationship between OSA and cognitive decline, or about whether these associations differ prospectively by *APOE* ε4 status, could not be drawn.

## 5 CONCLUSION

These limitations notwithstanding, the results of this study suggest that, in middle‐aged adults, OSA is associated with subtle yet measurable memory impairment and greater dementia risk, irrespective of *APOE* ε4 carriage. These findings also imply a dissociation between OSA‐related cognitive vulnerability and dementia risk in midlife, whereby subtle memory impairment is most evident in untreated OSA, while elevated dementia risk reflects cumulative vascular burden irrespective of treatment status. As such, routine screening for OSA in middle‐aged adults may help identify individuals at risk of dementia and provide an opportunity for intervention before clinically significant cognitive impairment emerges. Future randomized controlled trials are needed to investigate whether targeting OSA together with associated vascular comorbidities could potentially slow or prevent cognitive decline or reduce dementia risk in later life.

## AUTHOR CONTRIBUTIONS

**Gabriel T. Abdelmessih**: Conceptualization; formal analysis; methodology; writing—original draft; writing—review and editing. **Lisa Bransby**: Writing—review and editing. **Hannah Cummins**: Writing—review and editing. **Melinda L. Jackson**: Conceptualization; supervision; writing—review and editing. **Yen Ying Lim**: Conceptualization; funding acquisition; supervision; writing—review and editing.

## CONFLICT OF INTEREST STATEMENT

All authors have no relevant disclosures to report. Author disclosures are available in the Supporting Information.

## CONSENT STATEMENT

All participants provided informed consent via the HBP online platform prior to completing the online assessments.

## Supporting information




**Supporting Information**: alz71553‐sup‐0001‐SuppMat.docx

**Supporting Information**: alz71553‐sup‐0002‐SuppMat.pdf
