# Supplementary material for: Associations of self‐reported obstructive sleep apnea with cognition and dementia risk in cognitively unimpaired middle‐aged adults
Source: Alzheimers Dement. 2026 Jun 29;22(7):e71553. doi: 10.1002/alz.71553 (PMC13312299; doi:10.1002/alz.71553)
Supplement: Supplementary file 1 — Supporting Information: alz71553‐sup‐0001‐SuppMat.docx [file ALZ-22-e71553-s001.docx]

Supplementary material for: Associations of self-reported obstructive sleep apnoea with cognition and dementia risk in cognitively unimpaired middle-aged adults.

Gabriel T. Abdelmessih, Lisa Bransby, Hannah Cummins, Melinda L. Jackson, Yen Ying Lim.

Figure S1. Distributions of *z*-scores for the Cogstate Brief Battery attention and memory composites

Table S1. Computation of the modified CAIDE risk score

Table S2. Associations of OSA with cognition and dementia risk by OSA treatment status

Figure S1. Distributions of *z*-scores for the Cogstate Brief Battery attention and memory composites


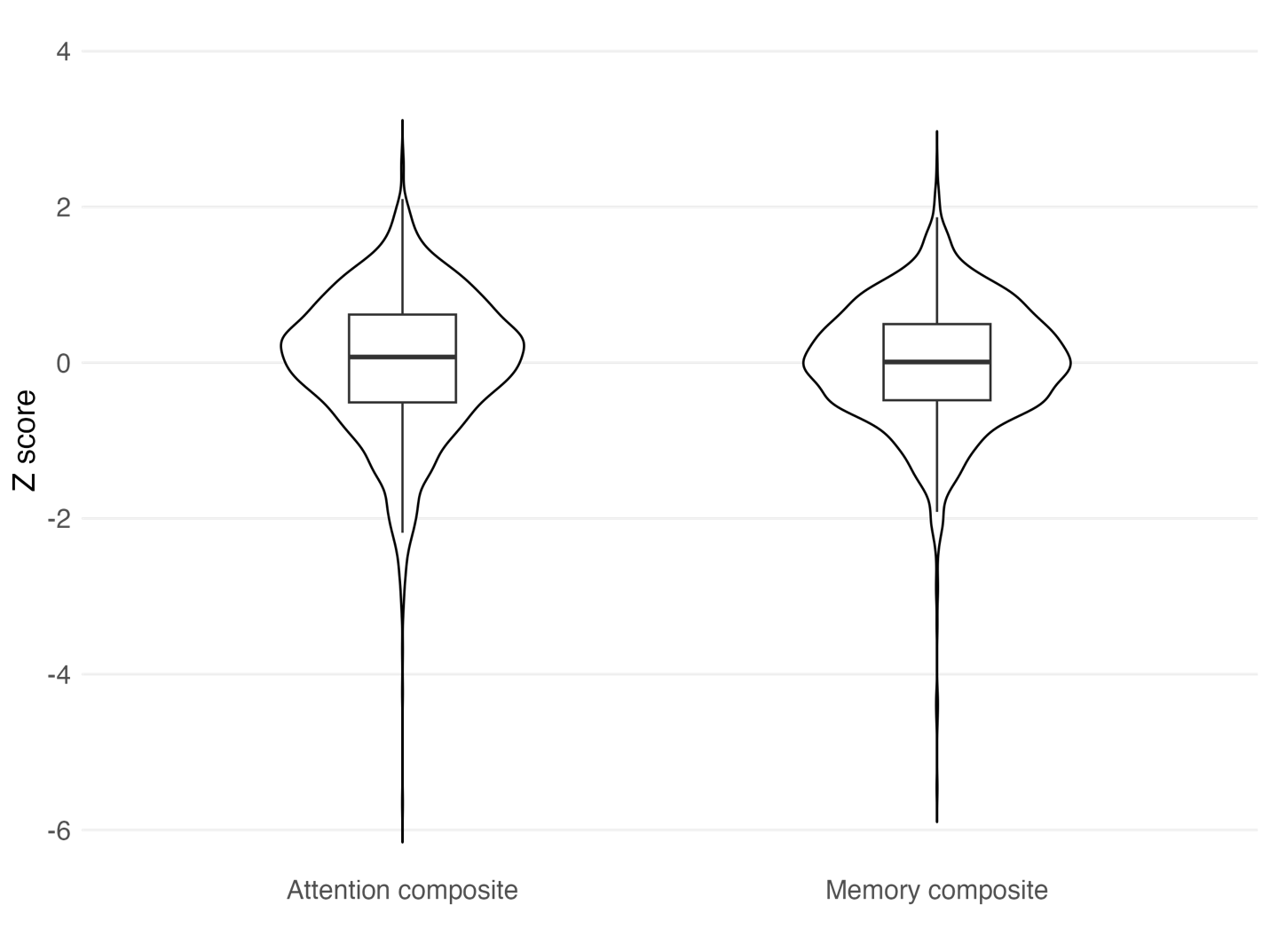


Table S1. Computation of the modified CAIDE risk score

| Factors | Original CAIDE Score (points) | Modified CAIDE Score (points) | Assessment Method |
| --- | --- | --- | --- |
| Age |  | - | Self-reported. |
| <47 years | 0 |  |  |
| 47-53 years | 3 |  |  |
| >53 years | 4 |  |  |
| Sex |  | - | Self-reported. |
| Female | 0 |  |  |
| Male | 1 |  |  |
| Education |  | - | Self-reported. |
| ≥10 years | 0 |  |  |
| 7-9 years | 2 |  |  |
| 0-6 years | 3 |  |  |
| Systolic blood pressure |  |  | Self-reported history of high blood pressure (yes/no). |
| ≤140 mmHg | 0 | 0 |  |
| >140 mmHg | 2 | 2 |  |
| BMI |  |  | Calculated from self-reported height and weight. |
| ≤30 kg/m^2^ | 0 | 0 |  |
| >30 kg/m^2^ | 2 | 2 |  |
| Cholesterol |  |  | Self-reported history of high cholesterol (yes/no). |
| ≤6.5 mmol/L | 0 | 0 |  |
| >6.5 mmol/L | 2 | 2 |  |
| Physical activity |  |  | Measured using the IPAQ. “High” and “Moderate” activity levels were scored as 0; “Low” activity levels were scored as 1. |
| Active | 0 | 0 |  |
| Inactive | 1 | 1 |  |
| Maximum Total score | 15 points | 7 points |  |

Abbreviations: BMI = body mass index; CAIDE = Cardiovascular Risk Factors, Ageing and Incidence of Dementia; IPAQ = International Physical Activity Questionnaire.

The original and modified CAIDE dementia risk scores were calculated for each participant by summing the relevant risk factor points. The original CAIDE score included both modifiable (e.g., systolic blood pressure, BMI, cholesterol, physical activity) and non-modifiable (e.g., age, sex, education) risk factors, with a maximum total score of 15. The modified CAIDE score included only modifiable risk factors, with a maximum total score of 7.

Table S2. Associations of OSA with cognition and dementia risk by OSA treatment status^*^

|  | ANCOVA | |  | EMM (SE) | | |  | Treated OSA+ versus OSA– | |  | Untreated OSA+ versus OSA– | |
| --- | --- | --- | --- | --- | --- | --- | --- | --- | --- | --- | --- | --- |
|  | *F* (df) | *P* value |  | OSA– | Treated OSA+ | Untreated OSA+ |  | Cohen’s *d* (95% CI) | *P* value |  | Cohen’s *d* (95% CI) | *P* value |
| Attention composite | 2.10 (2, 1824) | .12 |  | 0.02 (0.02) | 0.04 (0.11) | -0.12 (0.11) |  | -0.13 (-0.40, 0.14) | .84 |  | -0.02 (-0.23, 0.28) | .24 |
| Memory composite | 5.10 (2, 1785) | .007 |  | -0.00 (0.02) | -0.08 (0.10) | -0.27 (0.10) |  | -0.08 (-0.31, 0.16) | .45 |  | -0.27 (-0.51, 0.02) | .01 |
| CAIDE risk score | 57.21 (2, 2330) | <.001 |  | 5.08 (0.05) | 7.37 (0.24) | 6.71 (0.28) |  | 0.98 (0.77, 1.19) | <.001 |  | 0.70 (0.46, 0.94) | <.001 |
| Modified CAIDE risk score | 56.26 (2, 2327) | <.001 |  | 1.50 (0.04) | 3.07 (0.18) | 2.37 (0.21) |  | 0.93 (0.72, 1.14) | <.001 |  | 0.51 (0.27, 0.76) | <.001 |

Abbreviations: ANCOVA = analysis of covariance; CAIDE = Cardiovascular Risk Factors, Ageing and Dementia; CI = confidence interval; EMM = estimated marginal mean; OSA = obstructive sleep apnoea; SE = standard error.

^*^Models adjusted for age, sex, and education where appropriate. Sample sizes: Attention composite, *N* = 1830 (OSA– = 1703, treated OSA+ = 67, untreated OSA+ = 60); Memory composite, *N* = 1791 (OSA– = 1666, treated OSA+ = 66, untreated OSA+ = 59); CAIDE risk scores, *N* = 2333 (OSA– = 2173, treated OSA+ = 92, untreated OSA+ = 68).
